# Correction: Rivastigmine attenuates the Alzheimer's disease related protein degradation and apoptotic neuronal death signaling

**DOI:** 10.1042/BCJ20200754_COR

**Published:** 2022-06-08

The authors of the original article “Rivastigmine attenuates the Alzheimer's disease related protein degradation and apoptotic neuronal death signalling” (*Biochem J* (2021) **478** (7): 1435–1451; https://doi.org/10.1042/BCJ20200754.) would like to correct an error in Figure 4. The authors have identified an error in Figure 4 related to two statistical asterisks (*). As reported in the result section, the chymotrypsin activity of cortex and HP (hippocampus) regions was not significantly altered in STZ administered rats (Con vs. STZ), while by mistake the statistical marks were pasted on bars. The corrected Figure 4 is included in this Correction. The authors apologise for this oversight and any inconvenience it may have caused.

The corrected figure and its legend/equation is presented below. The authors declare that this Correction does not change the results or conclusions of the original paper.

**Figure 4.**
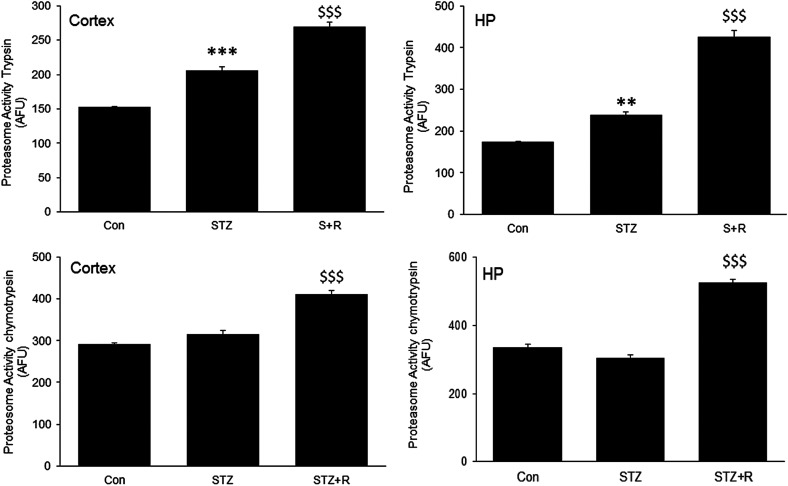
Effect of rivastigmine on proteasome activity. Bar diagram represents the proteasome activity of trypsin & chymotrypsin enzymes in both cortex & hippocampus regions of rat brain. Abbreviations: Con, control; R, Rivastigamine; STZ, streptozotocin. (n=6) Data are presented as mean±SEM analyzed by one way ANOVA post hoc Newman Keuls multiple comparison test, Control vs STZ — ** P<0.01, *** P<0.001; STZ vs STZ+R− $$$ P<0.001.

